# Ultrasonographic changes in fetal gastrointestinal motility during the last ten days before parturition in dogs

**DOI:** 10.3389/fvets.2022.1000975

**Published:** 2022-10-19

**Authors:** Giulia Siena, Stefano Romagnoli, Michele Drigo, Barbara Contiero, Francesca di Nardo, Chiara Milani

**Affiliations:** Department of Animal Medicine, Production and Health, University of Padova, Padua, Italy

**Keywords:** pregnancy, bitch, gastrointestinal motility, fetal development, peristalsis, gestational age, ultrasound

## Abstract

Fetal gastrointestinal motility (FGM) was suggested as useful to assess fetal maturity. Our study aimed to quantify FGM in relation to days before parturition (DBP), maternal size, and sex ratio of pups. During the last ten days of pregnancy, 23 clinically healthy pregnant bitches of 16 different breeds ranging in age from 2 to 9 years and body weight from 3.5 to 56.8 kg were monitored twice. The fetal intestine was observed in longitudinal and transversal scan on 3 of the most caudal fetuses in both uterine horns. Gestational age was counted backward from parturition day. The number of fetuses showing FGM was recorded in time in I (−11/−5 DBP) and II (−4/0 DBP). A Mann–Whitney test was performed to analyze variations of FGM% in relation to time and parity. A Kruskal–Wallis test was performed to identify variations of FGM% in relation to maternal size and sex ratio. Statistical significance was set at α = 0.05. A total of 147 FGM observations on 50 ultrasonographic monitoring points were performed. The FGM% was higher during time II compared to time I (median: 33%, range 0–100% vs. 100%, range 33–100%; *P* < 0.0001). FGM% was higher in small compared to large size bitches (median: 100%, range 67–100% vs. 67%, range 0–100%; *P* = 0.01). FGM% was not affected by parity and sex ratio. As previously reported, a significant increase in FGM% was observed in the last five DBP. FGM observation may be influenced by the maternal size, with easier evaluation in small size bitches, as well as ultrasound equipment and positioning.

## Introduction

The prediction of the canine parturition date is of primary importance in preventing neonatal death and ensuring assistance at parturition or planning a C-section. Many clinical, behavioral, and ultrasonographic parameters may be used at different stages of pregnancy (1–3). Fetal parameters such as inner chorionic cavity (ICC), crown-rump length (CRL), body diameter (BD), and biparietal diameter (BP) may be measured by ultrasound (US) throughout pregnancy. ICC is the most accurate fetal parameter in early pregnancy, whereas, in late pregnancy, the most accurate parameter is reported to be BP (1, 2, 4). CRL and BD are useful for evaluating gestational age in clinical practice (4, 5). Nowadays, many fetal parameters have been described and characterized as useful for the determination of the parturition day (4, 6–8) by calculating specific formulas, depending on the size of the bitch (4, 7, 9–12) and on the breed (13–16). However, all formulas based on fetal measurements are not accurate enough during the last week of pregnancy (17, 18). For this reason, predicting the parturition date when pregnant bitches are examined for the first time during this week is still challenging (1–3).

The US observation of fetal intestinal development is a useful tool for monitoring pregnancy progression, and the appearance of fetal gastrointestinal motility (FGM) indicates the conclusion of organogenesis. For this reason, FGM was suggested as a useful parameter to assess fetal maturity (19). Gil et al. (19) proposed four different developmental phases, starting from the first US visualization of the organ at 23–19 days before parturition (DBP). The first observation of FGM was reported in the third developmental phase from 13 to 9 DBP when FGM was visualized only in some intestinal portions after a prolonged US observation of the organ. During the fourth developmental phase (from 4 to 1 DBP), the intestinal wall layers, as well as dilation of some portions of the intestine and intraluminal mucous and fluid content, were easily evident. FGM was reported after a few seconds of US monitoring of the intestine (19).

In a previous study, we reported the daily observation regarding the percentage of fetuses with recognizable FGM (FGM%) during the last 10 days of pregnancy (from −9 to 0 DBP) (20). In that study, a weak negative correlation was found between FGM and vaginal temperature. In contrast, a correlation was not found between FGM and serum progesterone concentration, fetal heart rate, and rectal temperature. We also observed an increase in FGM% from 17.1 to 63.3% in the last 5 days prepartum (20). Assessing the magnitude and timely order of such an increase was complicated as the study was not designed to investigate differences in FGM over time as a repeated measure (each measure was not systematically performed with a standard frequency and time interval). Based on this study, we hypothesized that the observation of FGM may differ during the last 5 days of pregnancy compared to the previous 5 days.

Moreover, as described by others in the literature, we hypothesized that this parameter could be affected by factors other than the approaching of parturition day. Therefore, the present study aimed to quantify the amount of FGM in relation to DBP, considering and comparing two specific time intervals in which the US was performed twice in the same dam. The effect of maternal size and sex ratio of pups on FGM was also assessed.

## Materials and methods

This study was approved by the University of Padova Ethics Committee (Project nr. 69/2018), and written consent was obtained by the owner of each bitch. Twenty-three healthy pregnant bitches of 16 different breeds, ranging in age from 2 to 9 years and in weight from 3.5 to 52.2 kg, were included in the study (Table 1). Bitches were presented to the Veterinary Teaching Hospital of the University of Padova and enrolled for estrous or pregnancy monitoring from July 2020 to May 2021. No treatments were administered to induce estrus and/or ovulation. The bitches were monitored by US using an 8–5 MHz convex transducer connected to a US unit (Philips Affiniti 50G, Italy) in dorsal or lateral recumbency, following hair clipping and application of a contact US gel to the abdominal region. Each bitch was examined at least twice on two non-consecutive days during two intervals: −10/−5 (time I) and −4/0 (time II) DBP. A collection of reproductive history and a clinical examination were performed during each consultation. Moreover, a complete cell blood count (CBC) was performed at time II to monitor the health status of the bitch. The included bitches were monitored by US without any need for hospitalization.

**Table 1:** Bitch identification number (n.), breed, age, body weight (BW), maternal size, parity, litter size, number of male and female pups, the sex ratio of pups, and days of consultation (day 0 = day of parturition) of bitches included in the study.

**Bitch** **n**.	**Breed**	**Age** **(years)**	**BW** **(kg)**	**Size**	**Parity**	**Time I** **(DBP)**		**Time II** **(DBP)**		**Litter** **size**	**Male** **pups**	**Female** **pups**	**Sex ratio** **of pups** **(%)**
1	Whippet	5	17.5	Medium	Primiparous	−6		−2		7	3	4	57
2	Whippet	3	16.7	Medium	Primiparous	−10		−3		7	3	4	57
3	Labrador retriever	3	38	Large	Primiparous	−8		−4		12	6	6	50
4	Flat coated retriever	3	33.5	Large	Primiparous	−7		−2		7	5	2	29
5	Kurzhaar	2	33.2	Large	Primiparous	−6		−3		10	6	4	40
6	Bull terrier	3	21.6	Medium	Primiparous	−9		−4		6	3	3	50
7	Norfolk terrier	4	8.8	Small	Pluriparous	−7		−4	−1	3	1	2	67
8	Flat coated retriever	8	38.5	Large	Pluriparous	−9		−4		10	6	4	40
9	Dachshund	3	10.6	Small	Primiparous	−5		−2		5			
10	Crossbreed	7	12.1	Small	Pluriparous	−7		−3		5	4	1	20
11	Epagneul breton	4	20	Medium	Primiparous	−8		−2		7	3	4	57
12	Flat coated retriever	4	30.8	Medium	Primiparous			−3	0	9	3	6	67
13	Flat coated retriever	5	34.8	Large	Pluriparous	−7		−4		7	2	5	71
14	Australian shepherd	8	26	Medium	Primiparous	−11	−7	−4		7	4	3	43
15	Flat coated retriever	7	33.5	Large	Pluriparous	−8		−4		10	3	7	70
16	Australian shepherd	9	26.9	Medium	Pluriparous	−6		−1		9	6	3	33
17	Flat coated retriever	4	30.5	Medium	Pluriparous	−10	−6	−2		9	6	3	33
18	Flat coated retriever	3	36.5	Large	Primiparous	−5		−1		11	5	6	55
19	Bloodhound	4	52.2	Large	Primiparous	−6		−1		14	8	6	43
20	Pomeranian	3	3.5	Small	Primiparous	−10		−2	−1	2	0	2	100
21	Bouvier des Flandres	5	34.5	Large	Primiparous	−11	−5			10	5	5	50
22	Maremmano-abruzzese sheepdog	2	40.3	Large	Pluriparous	−8		−4		6	4	2	33
23	French bulldog	2	11.7	Small	Primiparous	−6		−2		5	2	3	60

Days of consultation are divided into two time intervals: −11/−5 (time I) and −4/0 (time II) days before parturition (DBP). The sex ratio of pups was calculated as the percentage of females present in each litter.

The days of monitoring were calculated based on ovulation day, when progesterone concentration was between 4–10 ng/mL (1, 21). Natural breeding or artificial inseminations (using fresh, chilled, or frozen semen) were performed based on the estimated ovulation day by our team or a referring veterinarian. When the ovulation day was not available, the days of monitoring were based on fetometry measures and formulas for calculating the parturition day reported in Table 2 (3, 6, 7, 22). Depending on the gestational period in which US monitoring was performed, the most suitable parameters were used, such as ICC (4, 7, 12), CRL (4, 6), BD (4, 6), and BP (4, 7, 12). The result was used to calculate gestational age or DBP using the related formulas described in the literature. These results were used to calculate the day on which US monitoring should be performed to assess FGM during the defined time ranges (Table 2). After parturition, gestational age was counted backward from the day of parturition (day 0), and the actual US monitoring day was confirmed based on this calculation.

**Table 2:** Formulas for calculation of days before parturition (DBP) or gestational age (GA: days after LH peak) in bitches of different sizes (small ≤ 10 kg, medium 11–25 kg, large ≥ 26 kg) (10–12) for the inner chorionic cavity (ICC), crown–rump length (CRL), body diameter (BD), and biparietal diameter (BP).

**Parameter**	**Maternal size**	**Formula**	**Reference**	**Time of** **measurement**
ICC	Small	DBP = (mm – 68.68)/1.53	(4, 7)	39–29 DBP
	Medium	DBP = (mm – 82.13)/1.8	(4, 7)	
	Large	DBP = (mm – 105.1)/2.5	(12)	
CRL	All sizes	GA = 24.64 + 4.54 × cm – 0.24 × cm^2^	(4, 6)	39–25 DBP
BD	All sizes	GA = 22.89 + 12.75 × cm – 1.17 × cm^2^	(4, 6)	29–7 DBP
BP	Small	DBP = (mm – 25.11)/0.61	(4, 7)	26–3 DBP
	Medium	DBP = (mm – 29.18)/0.7	(4, 7)	
	Large	DBP = (mm – 30)/0.8	(12)	

Measurements (scale either in mm or cm) are the mean of ICC, CRL, BD, and BP measurements in all measured fetuses. Time of measurement refers to the DBP at which each formula was used to estimate the parturition day. References are also reported.

Fetal intestine of the 3 most caudal fetuses in both uterine horns was observed for at least 30 seconds in a longitudinal and transversal scan. The number of fetuses showing FGM was recorded for each US monitoring point (videos available as Supplementary material). FGM was evaluated for each monitored fetus using a dichotomic score: absent when no bowel movements were evident during the US observation or present when evident intestinal peristalsis was assessed. The US observations were then reported as FGM% (percentage of fetuses showing FGM). The health of the examined pups was assessed up to 2 weeks of age.

Observing any abnormalities or clinical signs of systemic pathologies and administering hormonal interfering drugs (e.g., corticosteroids, sex hormones) during the 6 months before the estrous phase or pregnancy were considered exclusion criteria. Singleton pregnancies, fetal heart rate alterations with values lower than 160 bpm (23), fetuses with US evidence of morphological abnormalities, a stillbirth rate ≥ 30%, the neonatal death of the monitored fetuses or elective C-section, performed before serum progesterone drop (progesterone > 2 ng/ml) were also considered as exclusion criteria. For progesterone assay, the serum samples were analyzed with a fluorescence enzyme immunoassay (FEIA) method using an Automated Immunoassay Analyzer 360 (AIA^®^360, TOSOH Corp., Japan) (24). Therefore, bitches in which the US monitoring days were based on fetometry were excluded if, after delivery, the US monitoring days counted backward from the parturition day (day 0) were not in the defined time range.

Statistical analysis was performed using XLSTAT (2017.1.1.62936). Normality was checked using the Shapiro–Wilk test. A Mann–Whitney test was performed to analyze the variations of FGM% observed in the selected fetuses in relation to time intervals (time I–II) and the parity of dams (primiparous vs. pluriparous). A Kruskal–Wallis test was performed to identify variations of FGM% in relation to maternal size (small ≤ 10 kg, medium 11–25 kg, and large ≥ 26 kg) (10–12) and sex ratio of pups (percentage of females classified as ≤ 40%, 41–60%, or > 60%). Sex ratio classes were created using the mean ± ½ of the standard deviation. Data are reported as median and range; for all the analyses, the level of statistical significance was set at α = 0.05.

## Results

A total of 23 bitches were included in our study. The ovulation day was unknown in 8 out of 23 bitches. Based on parturition day, the duration of time interval I was extended backward by one day (from −11 to −5 DBP) as two bitches whelped one day later than expected. Twenty-one bitches were monitored during both time intervals. In two bitches, the two US monitoring points were performed only in one time interval (bitch n. 12 and 21 were monitored on days −3 and 0 and −11 and −5 before parturition, respectively). Nineteen bitches were monitored once for each time interval. In contrast, four bitches (n. 7, 14, 17, and 20) were monitored three times during the period of interest: two of them were monitored twice during time interval I, while the other two were monitored twice during time interval II. Five bitches were small size, while eight and ten were medium and large size, respectively. Fifteen bitches were primiparous, and eight were pluriparous (Table 1). Eleven bitches had natural parturition, whereas a C-section was performed in the other 12 cases. Litter size was in the interval of 2–14 pups. In 21 bitches, the US observation of FGM was performed on three of the most caudal fetuses in both uterine horns. In one bitch, US observations were made on two fetuses (as she carried a pregnancy of just two fetuses), and in one bitch on two out of three fetuses because the third one was excluded due to a US diagnosis of fetal anasarca. All the fetuses in which FGM was examined were healthy and alive at birth as well as 2 weeks after birth. The sex ratio of pups was reported in 22 out of 23 bitches. A total of 173 pups (85 females and 88 males) were born. In the monitored pregnancies, seven bitches were in the group carrying ≤ 40% of female pups, ten had a percentage of female pups in the interval of 41–60%, and five had > 60% of female pups.

A CBC was performed in 21 out of 23 bitches: on time I in two bitches and on time II in all the other 19 bitches. No abnormalities were found except for a high concentration of platelets (516^*^10^3^ /uL, range 173–800^*^10^3^ /uL), low hematocrit (38.2%, ranging between 32.7–46.3%), low concentration of hemoglobin (12.8 g/dL, range 11.1–15 g/dL), and an increase in the concentration of eosinophils (860 /uL, range 30–1970 /uL) (Table 3). No abnormal clinical signs were reported in any bitch.

**Table 3:** Cell blood count results: Bitch identification number (*n*.), days before parturition (DBP) in which the exam was performed, hematocrit (HCT, normal range of 38–49.5%), eosinophils (normal range of 130–530/uL), platelet (normal range of 211–384^*^10^3^/uL), hemoglobin (normal range of 13.3–17.2 g/dl), and white blood cells (WBC) (normal range of 8.53–16.61^*^103/μl).

**Bitch n**.	**DBP**	**HCT** **(%)**	**Eosinophils** **(/uL)**	**Platelet** (*10^3^/uL)	**Hemoglobin** **(g/dl)**	**WBC** (*103/μl)
1	−2	42	310	577	14.1	7.52
2	−3	42.8	100	464	14.6	9.08
3	−4	37.1	1,080	516	12.3	16.31
4	−2	36.8	1,020	653	12.4	10.39
5	−3	38.7	1,100	410	13.4	10.81
6	−4	46.3	50	393	15	14.88
7	−1	37.8	220	173	12.8	7.12
8	−4	34.7	1,930	258	11.5	14.05
9	−2	38.7	1,100	456	12.8	13.50
10	−3	37.7	860	480	12.7	11.07
11	−2	44	1,210	718	15	11.06
12	0	39.3	1,970	800	13	15.15
13	−4	32.7	1,720	743	11.2	13.59
14	−4	38.2	157	385	12.7	7.85
15	−4	34.4	870	647	11.5	13.76
16	−1	41	240	457	13.1	8.34
17	−6	40.4	1,520	541	13.7	11.44
18	−5	33.9	30	521	11.1	11.96
19	−1	35.9	670	535	12	15.08
20	−1	43.3	310	198	14.4	9.34
21	−5	34.2	260	621	11.4	11.45

**Figure 1:**
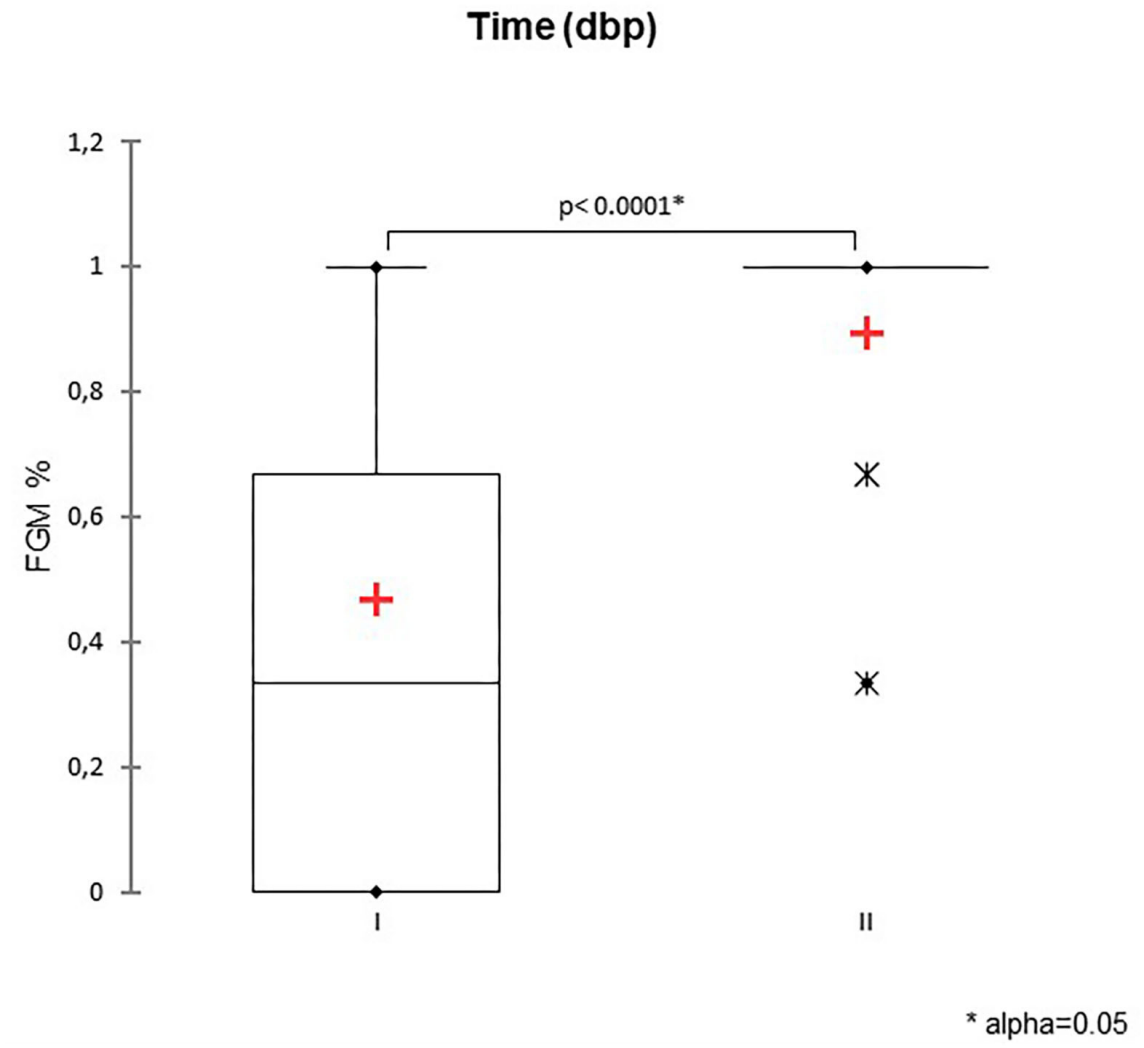
The box plot reports the percentage of fetuses showing fetal gastrointestinal motility (FGM%) during two-time intervals: time I −11/−5 days before parturition (DBP) and time II -4/0 DBP. Asterisk (^*^) indicates a statistically significant difference between classes (*P* < 0.0001). The mean is indicated as a red cross (+).

**Figure 2:**
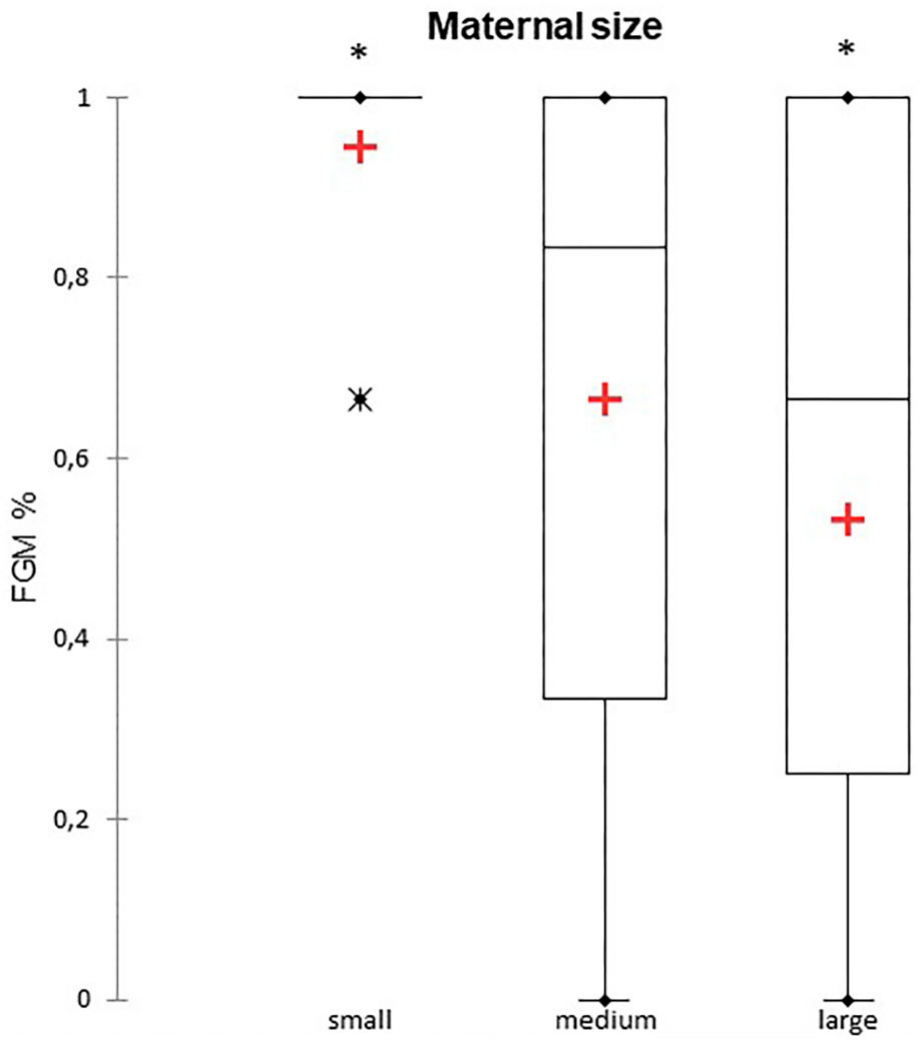
The box plot reports the percentage of fetuses showing fetal gastrointestinal motility (FGM%) in relation to maternal size in small (≤ 10 kg), medium (11–25 kg), and large size (≥ 26 kg) bitches (10–12). Asterisk (*) indicates a statistically significant difference between classes (*P* = 0.01). The mean is indicated as a red cross (+).

**Figure 3:**
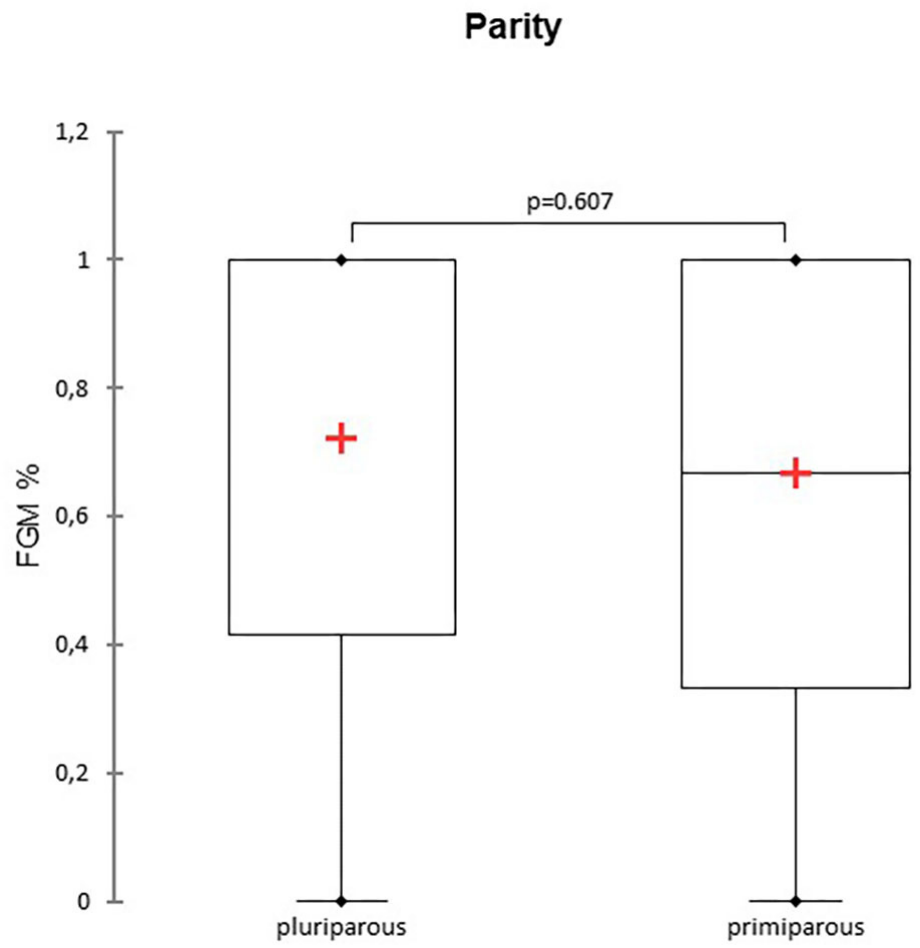
The box plot reports the percentage of fetuses showing fetal gastrointestinal motility (FGM%) in pluriparous and primiparous bitches. The mean is indicated as a red cross (+).

**Figure 4:**
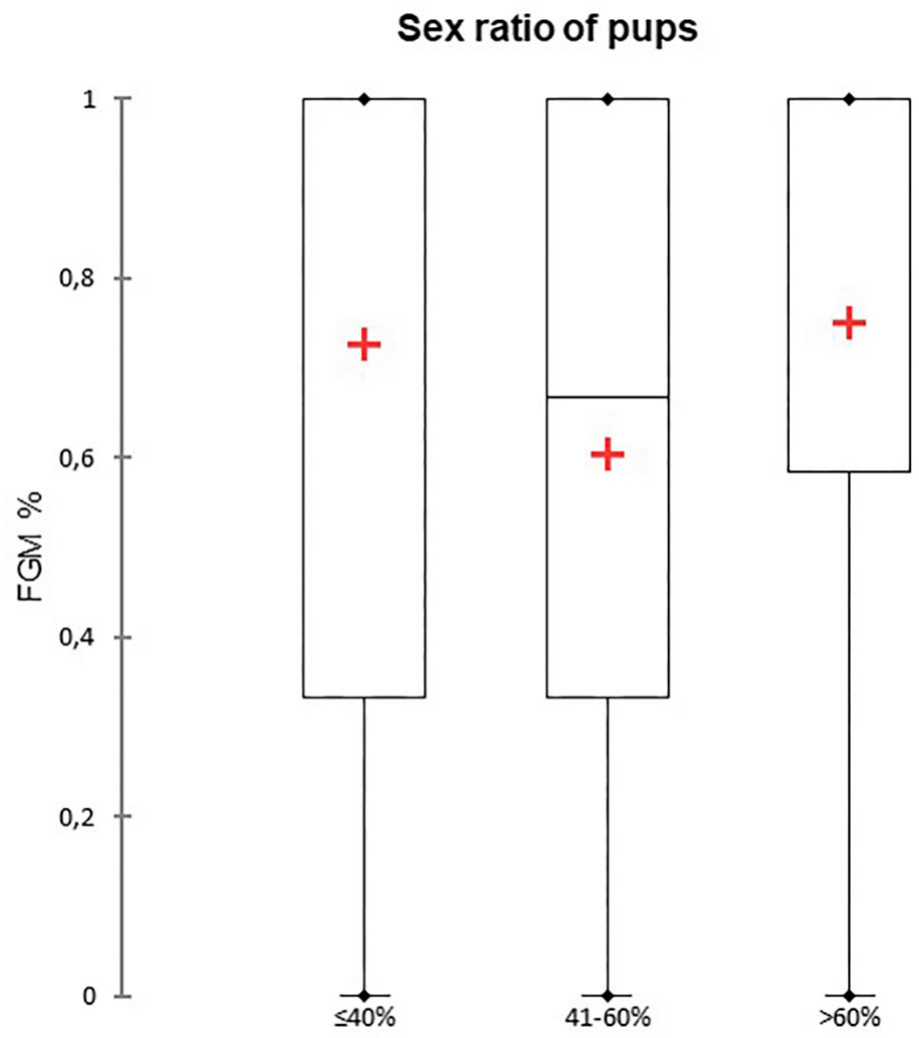
The box plot reports the percentage of fetuses showing fetal gastrointestinal motility (FGM%) dependent on the sex ratio of pups. Sex ratios were divided into three different classes depending on the percentage of females: ≤ 40%, 41–60%, and > 60%. The mean is indicated as a red cross (+).

A total of 147 FGM observations on 50 US monitoring points (25 on time I and 25 on time II) were performed. A significant difference for FGM% between time I and II was found, with a higher median FGM% during time II compared to time I (33%, range 0–100% vs. 100%, range 33–100%; *P* < 0.0001) (Figure 1). Considering maternal size, a higher FGM% was observed in small compared to large size bitches (100%, range 67–100% vs. 67%, range 0–100%; *P* = 0.01) (Figure 2). The FGM% was not affected by the parity of dams and sex ratio of pups (*P* = 0.607 and *P* = 0.419, respectively) (Figures 3, 4).

## Discussion

In the canine species, the US evaluation of fetal intestine development was performed by Gil et al. (19). They described for the first time four different phases of intestinal development detected by US. Pregnant bitches were divided into two groups, depending on the type of parturition (natural vs. C-section). Based on these two groups, each phase of intestinal development was defined with a specific time range. The day of the C-section was established based on fetal heart rate (< 160 bpm), which was used as a marker of fetal distress (emergency C-section) (19, 23). In our study, all C-sections were elective (based on progesterone concentration < 2 ng/ml); thus, we considered that our study was performed during the period described for natural parturition.

In an earlier study, we monitored FGM during the last 10 days of pregnancy (−9/0 DBP), describing a progressive increase in FGM% as parturition approaches. A marked increase (from 17.1% to 63.3%) was observed during the last ten DBP (20). In that study, each bitch was checked only once, which made it impossible to assess the significance of the increase in FGM% over time (20). Therefore, we decided to determine whether or not FGM% is higher during the last 5 days compared to the previous 5 days of pregnancy, as the last decade of gestation is a period in which accurate parameters to predict the delivery day are still lacking (1–4).

In our study, we observed a lower FGM% (33%) during time I (−11/−5 DBP) compared to 100% during time II (−4/0 DBP). Moreover, US detection of FGM was easier to observe in small compared to large size bitches during the same observation period. Maternal size may influence the ease of detection of FGM by the clinician, as it could be easier to observe fetal intestinal peristalsis when the size of the dam is small compared to large size. Furthermore, it should be considered that the US unit and probe characteristics, the dam's temperament, her positioning during the US, and the operator's experience influence the evaluation of FGM (25). Further studies with a larger sample size are needed to confirm a correlation between maternal size and the US detection of FGM in the canine species as well as to assess the importance of other environmental and managerial factors.

In our study, FGM% was not influenced by the sex ratio of pups. In human medicine, sex affects fetal maturity by acting as a regulatory factor for the surfactant system, influencing the Na+ transport channel expression and type II pneumonocyte maturation. The difference between male and female development seems to be related to sex hormones and sex-related genetic differences (26, 27). Moreover, in the canine species, fetal maturity is reported to be reached at different time points depending on fetal sex: female fetuses are mature at 59 days post-ovulation, whereas male fetuses mature one day later (28–30). In dogs, pregnancy length seems not to be affected by sex ratio when related to litter size (29).

In our study, no abnormalities were observed in CBC results except for those related to a physiological pregnancy at term (31, 32) and an increase in eosinophils, which may be due to the presence of intestinal parasites. Unfortunately, a fecal exam was not performed on the bitches of our study who showed an increase in eosinophils.

In horses, FGM is highly correlated with DBP, increasing the frequency of FGM observation as parturition approaches. A model was developed considering the combined use of FGM and a maternal parameter, such as tail head relaxation (33). In human medicine, FGM increases progressively from 16 to 40 weeks of pregnancy, and after week 28 FGM is more closely correlated to gestational age than BP or femur length (34). In humans, the combined use of FGM, intraluminal echogenicity of the colon, development of colonic haustra, and colon diameter may be used alone to estimate gestational age. These parameters are useful, especially during the last trimester when the US estimation of gestational age is less accurate than in the other pregnancy periods (34).

Further studies are needed to identify a panel of parameters useful for predicting parturition day during the last ten days of canine pregnancy. In human reproduction, the assessment of persistent US abnormalities in the fetal intestine (dilation or hyperechogenicity) is predictive of the presence of neonatal abnormalities (35). In canine reproduction, assessing FGM during the last decade of pregnancy could also be useful for assessing fetal gastrointestinal abnormalities.

In conclusion, in the pregnant bitches of our study, a marked increase in fetuses showing fetal gastrointestinal motility (from 33 to 100%) was observed in the last 5 days compared to the previous five days before parturition. Peristalsis is a useful tool to assess fetal maturity in the prepartum period in the bitch. Maternal size also influenced the detection of the percentage of fetuses showing fetal gastrointestinal motility, with higher values in small compared to large size bitches. Further studies are needed to assess whether fetal gastrointestinal motility may be used as the only parameter to plan a C-section in clinical practice. In the meantime, fetal gastrointestinal motility may be of help in the estimation of days before parturition as well as in deciding the best day to start monitoring prepartum progesterone, especially when ovulation day is unknown. Further studies are needed to better quantify this parameter variations in correlation with fetal maturity around parturition time.

## Data availability statement

The raw data supporting the conclusions of this article will be made available by the authors, without undue reservation.

## Ethics statement

The animal study was reviewed and approved by University of Padova Ethics Committee (Project nr. 69/2018). Written informed consent was obtained from the owners for the participation of their animals in this study.

## Author contributions

CM, MD, and SR: study conception. GS, CM, and FN: practical execution. BC and MD: analysis and interpretation of results. CM and SR: supervision. GS: writing—original draft. GS, SR, MD, BC, CM, and FN: editing. CM: funding acquisition. All authors read and approved the final manuscript.

## Funding

This research was funded by the Italian Ministry of University and Research (MUR, DOR 2025554/20).

## Conflict of interest

The authors declare that the research was conducted in the absence of any commercial or financial relationships that could be construed as a potential conflict of interest.

## Publisher's note

All claims expressed in this article are solely those of the authors and do not necessarily represent those of their affiliated organizations, or those of the publisher, the editors and the reviewers. Any product that may be evaluated in this article, or claim that may be made by its manufacturer, is not guaranteed or endorsed by the publisher.
