# Supplementary material for: Ultrasonographic changes in fetal gastrointestinal motility during the last ten days before parturition in dogs
Source: Front Vet Sci. 2022 Oct 19;9:1000975. doi: 10.3389/fvets.2022.1000975 (PMC9628212; doi:10.3389/fvets.2022.1000975)
Supplement: Supplementary file 1 [file Presentation_1.pptx]

## Slide 1
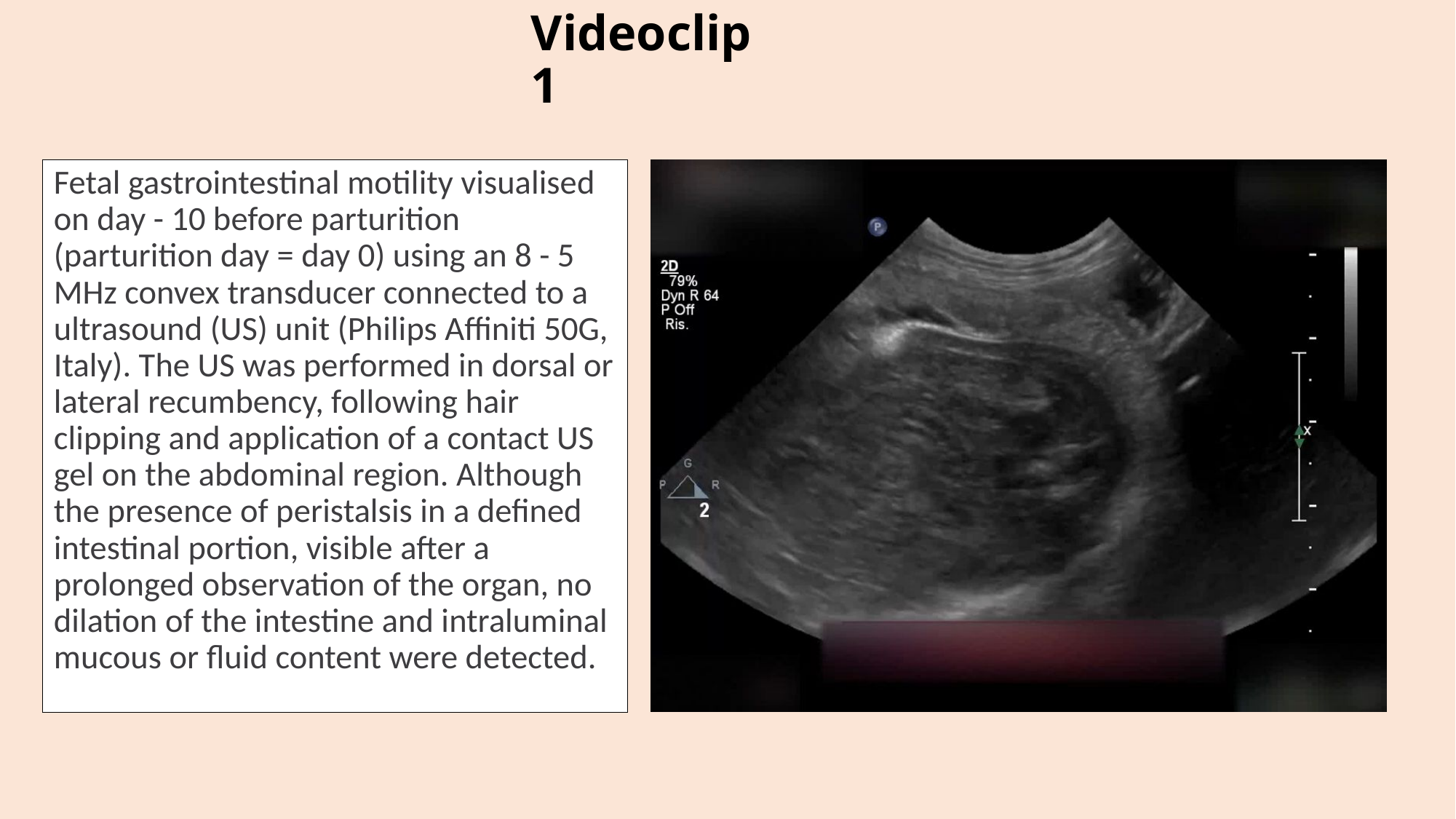

# Videoclip 1
Fetal gastrointestinal motility visualised on day - 10 before parturition (parturition day = day 0) using an 8 - 5 MHz convex transducer connected to a ultrasound (US) unit (Philips Affiniti 50G, Italy). The US was performed in dorsal or lateral recumbency, following hair clipping and application of a contact US gel on the abdominal region. Although the presence of peristalsis in a defined intestinal portion, visible after a prolonged observation of the organ, no dilation of the intestine and intraluminal mucous or fluid content were detected.

## Slide 2
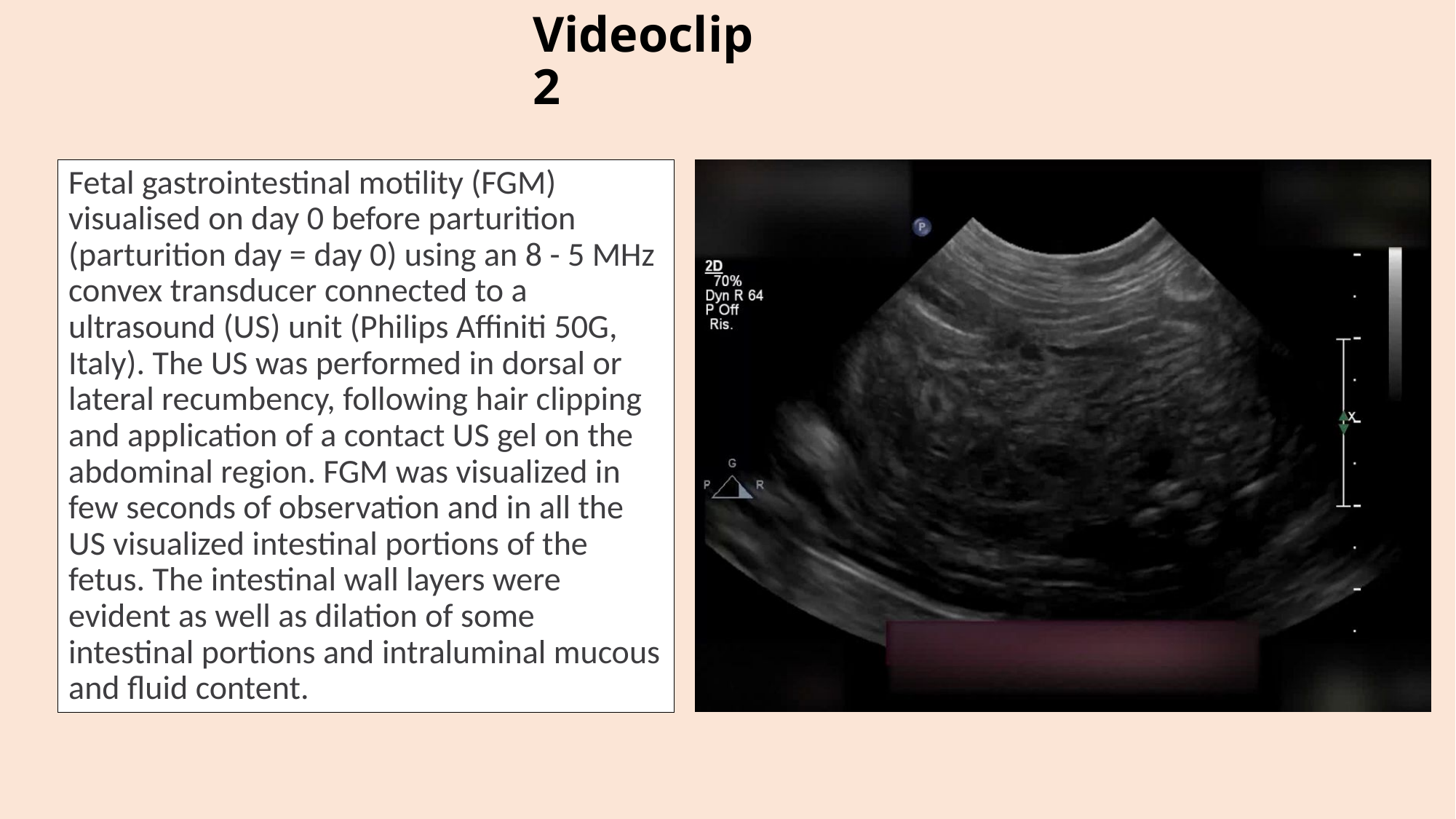

# Videoclip 2
Fetal gastrointestinal motility (FGM) visualised on day 0 before parturition (parturition day = day 0) using an 8 - 5 MHz convex transducer connected to a ultrasound (US) unit (Philips Affiniti 50G, Italy). The US was performed in dorsal or lateral recumbency, following hair clipping and application of a contact US gel on the abdominal region. FGM was visualized in few seconds of observation and in all the US visualized intestinal portions of the fetus. The intestinal wall layers were evident as well as dilation of some intestinal portions and intraluminal mucous and fluid content.
